# *Notes from the Field:* Increase in Nontoxigenic *Corynebacterium diphtheriae* **—** Washington, 2018**–**2023

**DOI:** 10.15585/mmwr.mm7317a4

**Published:** 2024-05-02

**Authors:** Amy G. Xie, Kyle Yomogida, Isha Berry, Nicole L. Briggs, Precious Esie, Arran Hamlet, Keely Paris, Erin Tromble, Chas DeBolt, Nicholas R. Graff, Eric J. Chow

**Affiliations:** ^1^Public Health – Seattle & King County, Seattle, Washington; ^2^Epidemic Intelligence Service, CDC; ^3^Washington State Department of Health; ^4^Division of Bacterial Diseases, National Center for Immunization and Respiratory Diseases, CDC; ^5^Division of Allergy and Infectious Diseases, Department of Medicine, University of Washington, Seattle, Washington; ^6^Department of Epidemiology, University of Washington, Seattle, Washington.

**Summary**

**What is already known about this topic?**

*Corynebacterium diphtheriae* infections can be caused by toxigenic and nontoxigenic strains. Diphtheria toxoid–containing vaccines (DTaP, Tdap, Td) only protect against toxigenic strains. Nontoxigenic *C. diphtheriae* infections are most frequently associated with cutaneous disease and are not vaccine preventable.

**What is added by this report?**

Review of all Washington nontoxigenic *C. diphtheriae* cases during a 5-year period revealed that infection prevalence is increasing. Unstable housing and recent illicit substance use were prevalent among patients. Severe disease can manifest as endocarditis and bacteremia.

**What are the implications for public health practice?**

Future nontoxigenic *C. diphtheriae* studies focusing on understanding treatment indications and effectiveness and characterizing modifiable risk factors and barriers to quality wound care might identify opportunities to implement strategies for reducing community spread of *C. diphtheriae*.

Toxin-producing *Corynebacterium diphtheriae*, an aerobic Gram-positive coccobacillus, is the predominant causative agent of diphtheria and is responsible for substantial morbidity worldwide (*1*). Infection with nontoxigenic *C. diphtheriae* is also associated with disease, but little is known about the clinical spectrum of illness or the incidence of nontoxigenic *C. diphtheriae* infections in the United States (*2*). Toxin gene acquisition and expression by nontoxigenic *C. diphtheriae* is biologically plausible and could lead to reintroduction of diphtheria into the United States, where diphtheria is no longer endemic (*3*). Understanding diseases caused by nontoxigenic forms of *C. diphtheriae* is important because diphtheria toxoid–containing vaccines create immunity to the toxin itself but cannot protect against infection or illness caused by nontoxigenic strains. In the state of Washington, detection of *C. diphtheriae* in any clinical specimen is immediately notifiable (*4*). Beginning in 2000, Washington mandated submission of all *C. diphtheriae* isolates to Washington State Public Health Laboratories (WSPHL). The number of reported nontoxigenic *C. diphtheriae* isolates in Washington has increased approximately tenfold, from 17 during 2012–2017 to 179 during 2018–2023; most infections occurred among King County residents. In November 2023, Washington State Department of Health, Public Health – Seattle & King County, and CDC conducted a statewide investigation of nontoxigenic *C. diphtheriae* cases to determine factors contributing to this increase and to describe the epidemiology of nontoxigenic *C. diphtheriae* and clinical characteristics of patients with nontoxigenic *C. diphtheriae* infections in Washington.

## Investigation and Outcomes

During January 1, 2018–September 30, 2023, *C. diphtheriae* isolates from 176 patients were identified in 14 (36%) of 39 Washington counties; all isolates were identified as *C. diphtheriae* at WSPHL and subsequently determined to be nontoxigenic by CDC. A public health team abstracted patient data^§^ from medical charts. Descriptive statistics were calculated using R software (version 4.3.1; R Foundation). This activity was reviewed by CDC, deemed not research, and was conducted consistent with applicable federal law and CDC policy.^¶^

Chart abstraction was conducted for 166 (94%) patients; 120 (72%) were male, and the median age was 44 years (range = 8 months–76 years) (Table). Among these patients, 171 nontoxigenic *C. diphtheriae* isolates were identified, including 134 (78%) from cutaneous wound culture; 130 (97%) of these cultures yielded polymicrobial results. However, *C. diphtheriae* was also isolated from blood (21; 12%) and other body fluids (16; 9%), including urine, sputum, and synovial fluid. Persons experiencing unstable housing (64%) or who recently** used illicit substances^††^ (63%) were disproportionately represented among patients. Lifetime injection drug use was only documented in 43% of patients and 40% of patients with cutaneous infections. Six patients (4%) received a diagnosis of endocarditis attributable to *C. diphtheriae* alone. Fourteen (8%) patients died from any cause during the study period. No patient had clinical findings suggestive of diphtheria.

**TABLE:** Demographic and clinical characteristics of patients with nontoxigenic *Corynebacterium diphtheriae* infection (N = 166) — Washington, 2018–2023

Characteristic	No. (%)
**Median age, yrs (range)**	43.9 (0.7–75.9)
**Sex**	
Female	45 (27.1)
Male	120 (72.3)
Unknown	1 (0.6)
**Medical history**	
History of hepatitis C infection	56 (33.7)
Venous stasis or insufficiency	28 (16.9)
Previous abscesses	28 (16.9)
Previous diagnosis of sepsis	21 (12.7)
Diabetes mellitus	19 (11.4)
Chronic kidney disease	8 (4.8)
HIV	7 (4.2)
Heart failure	7 (4.2)
Cardiac valve disease	5 (3.0)
Cirrhosis	5 (3.0)
**Housing**	
Currently experiencing homelessness	106 (63.9)
Previously experienced homelessness	27 (16.3)
Stable	12 (7.2)
Unknown	21 (12.7)
**Drug use**	
Recent illicit substance use*	104 (62.7)
Lifetime IV drug use	72 (43.4)
**Specimen source (N = 171 isolates)^†^**	
Wound	134 (78.4)
*C. diphtheriae* only^§^	3 (2.2)
Polymicrobial^§,¶^	130 (97.0)
Unknown^§^	1 (0.6)
Blood	21 (12.3)
*C. diphtheriae* only**	11 (52.4)
Polymicrobial^§,^**	10 (47.6)
Other body fluid^††^	16 (9.4)
**Setting**	
Emergency department	123 (74.1)
Primary care	15 (9.0)
Urgent care	12 (7.2)
Other	16 (9.6)
**Outcomes and complications**	
Deceased during study period (2018–2023)^§§^	14 (8.4)
Hospitalization in ICU during encounter when *C. diphtheriae* infection was diagnosed	11 (6.7)
Bacteremia with *C. diphtheriae*	21 (12.7)
Endocarditis caused by *C. diphtheriae*^¶¶^	6 (3.6)

**Abbreviations:** ICU = intensive care unit; IV = intravenous.

* Recent use refers to the 90 days preceding the encounter when *C. diphtheriae* was isolated. Illicit substances include amphetamines, opiates, and psychoactive recreational drugs and does not include alcohol, tobacco, or marijuana.

^†^ Some patients had more than one specimen collected.

**^§^** Percentage of 134 wound cultures.

**^¶^** Wound culture with *C. diphtheriae* and at least one other organism.

** Percentage of 21 blood cultures.

**^††^** Other isolates were from urine, sputum, or synovial fluid.

^§§^ Death attributable to any cause (not limited to infection).

^¶¶^ Clinical diagnosis of endocarditis in addition to monomicrobial *C. diphtheriae* blood culture.

Laboratory directors from five clinical laboratories that had processed 65% of the total *C. diphtheriae* isolates were interviewed about protocols for identifying gram-positive bacilli. Most reported increasing use of matrix-assisted laser desorption/ionization time-of-flight (MALDI-TOF) mass spectrometry, which can identify unknown molecules from a robust database of common patterns. The laboratory that identified the largest proportion of *C. diphtheriae* isolates in Washington (56; 34%) has not changed microbiological techniques or protocols for identifying *C. diphtheriae* since 2013, when MALDI-TOF was implemented.

## Preliminary Conclusions and Actions

Although the clinical characteristics of nontoxigenic *C. diphtheriae* infections are distinct from those of diphtheria caused by toxin-producing *C. diphtheriae* strains, nontoxigenic *C. diphtheriae* infection can be associated with severe disease; in this analysis, 74% of patients were initially evaluated in an emergency department, 12% had bacteremia, and 4% had endocarditis. Presentation of illness was consistent with infections caused by other organisms and recognized as *C. diphtheriae* only when cultures resulted. Recognizing *C. diphtheriae* is important because it is associated with morbidity and mortality. Fourteen (8%) patients died soon after detection of nontoxigenic *C. diphtheriae* infection; causes of death varied and were affected by factors that included underlying medical conditions, infections, experience of homelessness, and substance use.

The stability of laboratory procedures in place since 2013 suggests that the increase in *C. diphtheriae* in Washington is likely not due to changes in laboratory techniques or protocols. The findings from this investigation are consistent with those from a 2011 Canadian study of 33 patients with cutaneous infections caused by nontoxigenic *C. diphtheriae* during 1998–2007; those infections primarily affected vulnerable populations experiencing unstable housing (*5*). Further investigations of reasons for the increase in nontoxigenic *C. diphtheriae* infections, including an assessment of risk factors for severe outcomes, could help identify opportunities to implement strategies to reduce community spread of *C. diphtheriae*.

## Footnotes

§ Demographic characteristics, housing status, substance use information, medical comorbidities, clinical management and illness course, and health care use patterns.

¶ 45 C.F.R. part 46, 21 C.F.R. part 56; 42 U.S.C. Sect. 241(d); 5 U.S.C. Sect. 552a; 44 U.S.C. Sect. 3501 et seq.

** Recent use of substances refers to the 90 days leading up to the encounter when *C. diphtheriae* was isolated.

†† Illicit substance use includes amphetamines, opiates, and psychoactive recreational drugs and does not include alcohol, tobacco, or marijuana.
